# Glycol Derived Carbon- TiO_2_ as Low Cost and High Performance Anode Material for Sodium-Ion Batteries

**DOI:** 10.1038/srep43895

**Published:** 2017-03-03

**Authors:** Hongwei Tao, Min Zhou, Kangli Wang, Shijie Cheng, Kai Jiang

**Affiliations:** 1 State Key Laboratory of Advanced Electromagnetic Engineering and Technology, School of Electrical and Electronic Engineering AND State Key Laboratory of Materials Processing, and Die & Mould Technology, School of Materials Science and Engineering, Huazhong University of Science and Technology, Wuhan 430074, P. R. China

## Abstract

Carbon coated TiO_2_ (TiO_2_@C) is fabricated by a convenient and green one-pot solvothermal method, in which ethylene glycol serve as both the reaction medium and carbon source without the addition of any other carbon additives. During the solvothermal process, ethylene glycol polymerize and coordinate with Ti^4+^ to form the polymeric ligand precursor, then the polymer brushes carbonize and convert to homogeneous carbon layer firmly anchored on the TiO_2_ nanoparticles (~1 nm thickness). The polymerization and carbonization process of the ethylene glycol is confirmed by FT-IR, Raman, TG and TEM characterizations. Benefiting from the well-dispersed nanoparticles and uniform carbon coating, the as-prepared TiO_2_@C demonstrate a high reversible capacity of 317 mAh g^−1^ (94.6% of theoretical value), remarkable rate capability of 125 mAh g^−1^ at 3.2 A g^−1^ and superior cycling stability over 500 cycles, possibly being one of the highest capacities reported for TiO_2_.

The development of advanced energy storage technology is of great importance to address the increasingly global concerns of energy shortage and environmental issues^1^,^2^. To date, Li-ion batteries (LIBs) represent the state-of-the-art technology due to their high energy density and have dominated the energy storage market of portable electronic devices. However, the limited lithium resources and high price of lithium-based compounds remains an obstacle for their expanded application in large-scale energy storage^3^,^4^. In contrast to lithium, sodium is widely distributed around the world and has a suitable redox potential (E^0^_Na/Na_^+^ = −2.71 V vs SHE), only 0.3 V above that of lithium. Sodium ion batteries (SIBs) with low cost and high efficiency seem to be the ideal alternative to LIBs, especially for grid-scale energy storage applications^5^,^6^,^7^.

Unfortunately, the large radius of sodium ions make it difficult to find appropriate Na-storage electrode materials with high capacity and rapid kinetics. With respect to the anode side, various types of hard carbon have been revealed to deliver considerable reversible capacity, but their low potential and large polarization raise safety issues for the practical battery applications^8^,^9^,^10^. Metallic anodes, such as Sn and Sb-based materials, have attracted significant attention due to their high Na-storage capacity^11^,^12^,^13^. However, the unavoidable volume change of these materials during the repeated sodiation/desodiation hinders their further applications. As a result, exploiting better anode materials with low cost and cycling stability is still necessary for the development of practically viable SIBs.

Titanium-based materials, a cost effective, structurally stable and sustainable material, is considered to be promising Na-storage anode^14^,^15^. Among various polymorphs of Ti-based anode materials, anatase TiO_2_ exhibit much better electrochemical performances owning to the three dimensional open structure, which is favorable for the Na^+^ transport and storage^16^. Nevertheless, the intrinsic low conductivity of pure TiO_2_ leads to low realizable capacity and poor rate performance. Considering that the electrochemical performances of TiO_2_ electrode is strongly depend on the morphology and pore size of the particles, varieties of TiO_2_ nanostructures have been designed and investigated as Na-storage materials with enhanced reversible Na-storage capacity, such as nanoparticles, nanotubes, nanorods, nanospheres, nanofibers^17^,^18^,^19^,^20^,^21^,^22^,^23^. However, the side reactions and structure instability of nanoparticles lead to low initial coulombic efficiency and poor cycling performances. Another effective strategy to increase the capacity utilization of TiO_2_ is heteratomic doping^24^,^25^,^26^,^27^. Doping elements with low charge states can create structure defects in the bulk TiO_2_ and thus enhance the electrical and ionic conductivities. Of significance, Pan *et al*.^26^ prepared Ni^2+^ doped TiO_2_ nanotubes with a maximum capacity of 286 mAh g^−1^ after 100 cycles at a current density of 50 mA g^−1^. However, the initial coulombic efficiency (CE), which are critical for practical SIBs, still need to be upgraded considerably. In attempt to further improve the electrochemical performances of TiO_2_, efforts have been devoted to combine the nanosized TiO_2_ with conductive carbon^28^,^29^,^30^,^31^,^32^. Carbon coating can provide conducting network and stabilize the SEI formation by restraining sodium ions, thus resulting in improved capacity utilization as well as initial coulombic efficiency and rate performances. Recently, Yang *et al*. reported a graphene supported TiO_2_ nanospheres with a superior Na storage capacity of 300 mAh g^−1^ at 20 mA g^−1^ and a high rate capability of 123.1 mAh g^−1^ at a high rate of 4.0 A g^−1^. Nevertheless, the long-term cycling stability of this material still need to be improved. Moreover, the high cost, low initial coulombic efficiency and complex synthesis route of graphene create a barrier for the large-scale applications of TiO_2_ anode.

Glycol is the common used reaction medium for the preparation of TiO_2_ with the advantages of effectively controlling the morphology and particle size^33^,^34^,^35^,^36^. In this work, we present a simple and green one-pot solvothermal method to fabricate carbon-coated TiO_2_ nanoparticles (TiO_2_@C), in which ethylene glycol serve as both the reaction medium and carbon source without the addition of any other carbon additives. During the solvothermal process, ethylene glycol polymerize and coordinate with Ti^4+^ to produce polymeric ligand precursor. Then in the subsequent annealing process, the polymer brushes pyrolyze and convert to a uniform and homogenous carbon layer firmly anchored on the surface of TiO_2_ nanoparticles. As expected, the as-prepared TiO_2_@C demonstrate a high reversible capacity of 317 mAh g^−1^ at 0.05 A g^−1^, strong rate capability of 125 mAh g^−1^ at 3.2 A g^−1^ and superior cycling stability over 500 cycles, offering a low cost and high performance anode material for SIBs.

## Results and Discussion

**Figure 1:**
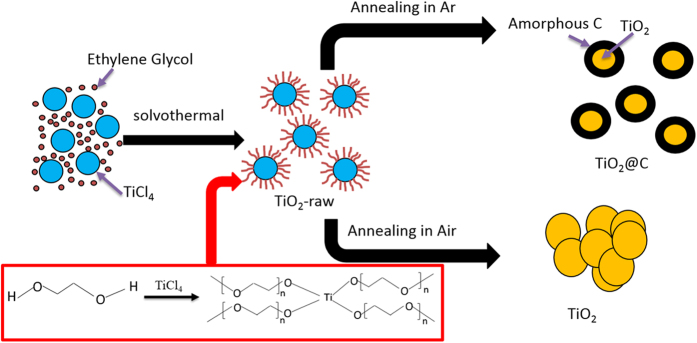
Schematic illustration of the synthesis of TiO_2_@C nanoparticles.

Figure 1 presents the typical synthesis route of the TiO_2_@C (TiO_2_) nanoparticles. The solvent (ethylene glycol) polymerized during the first solvothermal process (equation 1). As polyethyleneglycol is rich in –OH and –C–O–C– groups, it can easily coordinate with Ti^4+^ to firmly anchor the precursor molecules on their surfaces (equation 2). The FT-IR spectrum of the TiO_2_-raw material reflects all the characteristic absorptions of the typical precursor, confirming the polymerization and coordination reaction mechanism stated above (Fig. S1). In the final annealing process, the polymer brushes carbonize and convert to uniform and homogeneous carbon coating on the TiO_2_ nanoparticles (equation 3). It is worth noted that the ethylene glycol cannot polymerize in the same condition without the presence of TiCl_4_, indicating that the Ti^4+^ play a catalysis role in the polymerization of ethylene glycol.


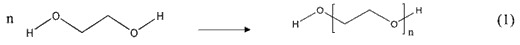



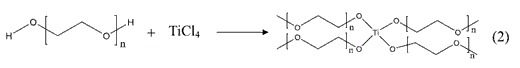



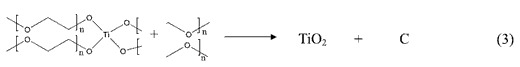


**Figure 2:**
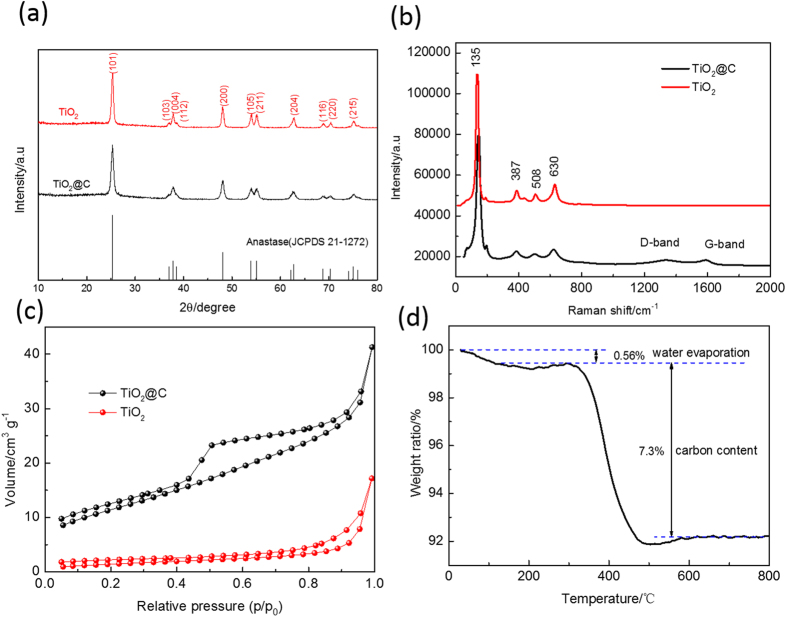
Physical characterizations of the TiO_2_@C and TiO_2_: (**a**) XRD pattern, (**b**) Raman spectra and (**c**) N_2_ adsorption–desorption isotherm of TiO_2_@C and TiO_2_; (**d**) TGA curve of TiO_2_@C.

The crystalline structure of the TiO_2_@C and TiO_2_ are examined by X-ray diffraction spectrometry (XRD). As shown in Fig. 2a, all the diffraction peaks of TiO_2_@C and TiO_2_ can be well indexed to the anatase phase TiO_2_ (JCPDS: 21-1272) with the tetragonal crystal structure belonging to *I4*_*1*_*/amd* space group, a = 3.784 ± 0.002 Å, and c = 9.514 ± 0.004 Å. The peak intensity of TiO_2_@C is weaker than that of TiO_2_, indicating the TiO_2_ nanoparticles embedded in amorphous carbon matrix. Based on the Debye Scherrer equation, the crystal sizes of TiO_2_@C and TiO_2_ are calculated to be ~24 nm and 27 nm, respectively. The X-ray photoelectron spectroscopy (XPS) spectra are recorded to analyze the chemical state of the TiO_2_@C. As shown in Fig. S2, there are two peaks of binding energies at 459 and 465 eV ascribed to Ti^4+^ 2p3/2 and Ti^4+^ 2p1/2 in the spectrum of the TiO_2_@C, suggesting the formation of TiO_2_. Raman spectra are recorded to investigate their surface composition and structures (Fig. 2b). The vibrational peaks at 135, 387, 508 and 630 cm^−1^ are observed in the Raman spectra of both TiO_2_@C and TiO_2_, well consistent with that of previously reported^37^,^38^. Besides, compared with TiO_2_, two extra characteristic peaks located at 1337 and 1587 cm^−1^ are detected in the Raman spectra of TiO_2_@C, corresponding to disorder carbon (D-band) and graphite carbon (G-band) attributed by the amorphous carbon coating layer^39^. The Brunauer–Emmett–Teller (BET) measurement is carried out to investigate the surface and porous structures of TiO_2_. As shown in Fig. 2c, N_2_ adsorption-desorption isotherms of TiO_2_@C and TiO_2_ can be identified as type IV isotherm (IUPAC), suggesting the mesoporous structure. According to the BET analysis, the specific surface areas of the TiO_2_ and TiO_2_@C are measured to be 5.5 and 41.3 m^2^ g^−1^, respectively. The large surface area and porous structure can not only increase the electrolyte/electrode contact areas, but also facilitate the kinetics of Na^+^ insertion/extraction and diffusion. Figure 2d presents the thermogravimetry analyses (TGA) curves of TiO_2_@C, the carbon contents in the TiO_2_@C is evaluated to be ~7.3 wt%.

**Figure 3:**
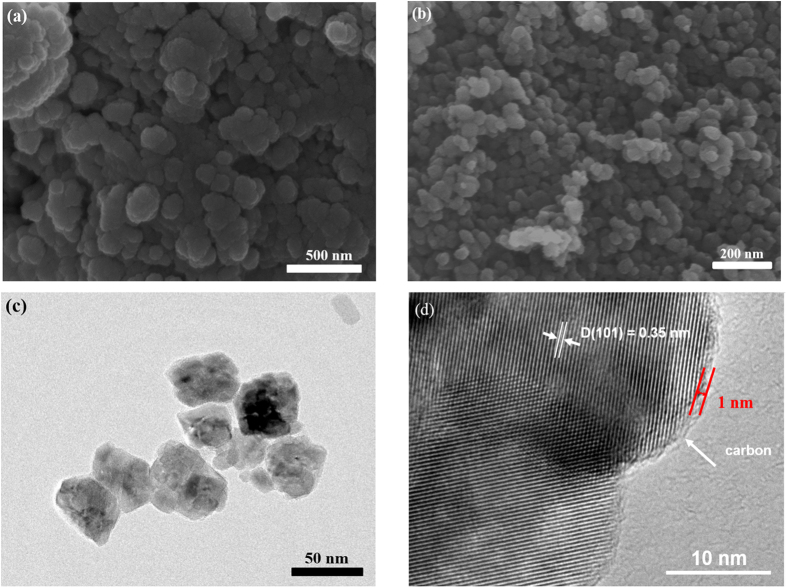
Morphological features of the TiO_2_@C and TiO_2_ particles: (**a**) SEM image of TiO_2_; (**b**) SEM image of TiO_2_@C; (**c**) TEM images and (**d**) high resolution TEM image of TiO_2_@C.

The as-prepared TiO_2_@C is in the form of black powders, while the color of the pure TiO_2_ is white (Fig. S3). The morphologies of the TiO_2_ and TiO_2_@C are presented in Fig. 3. As shown in Fig. 3a, the as-prepared TiO_2_ appeared as uneven particles with the average particle size of ~200 nm, which consist of aggregated primary crystallites with the crystal size of ~25 nm (Fig. S4). In contrast, the TiO_2_@C emerges as well-dispersed nanoparticles with much smaller size ranging from 30 to 50 nm (Fig. 3b,c), indicating the carbon coating can prevent the TiO_2_ nanoparticles from aggregating. The high-resolution TEM image of TiO_2_@C (Fig. 3d) reveals clear lattices with spacing of 0.35 nm, corresponding to the [101] planes of anatase TiO_2_, in accordance with the XRD result. Besides, it is clearly visualized that the amorphous carbon shell with the thickness of ~1 nm are well decorated on the surface of the TiO_2_ nanoparticles, ensuring the high conductivity and stable structure of the TiO_2_@C composites.

**Figure 4:**
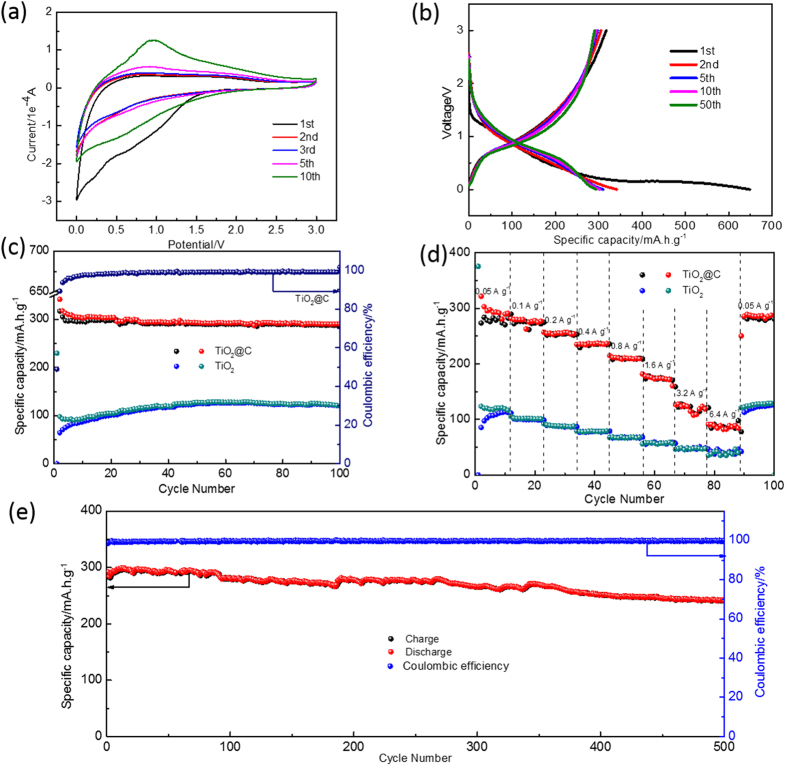
Electrochemical characterizations of the TiO_2_@C electrode: (**a**) CV curves obtained at a scan rate of 0.5 mV s;^−1^ (**b**) charge-discharge profiles at a current rate of 0.05 A g^−1^ in the first 50 cycles; (**c**) cycling performance at a constant current of 0.05 A g;^−1^ (**d**) rate capability at various current rates from 0.05 A g^−1^ to 6.4 A g;^−1^ (**e**) long-term cycling performances at a constant current density of 400 mA g^−1^.

The electrochemical reactivity of the TiO_2_@C sample is investigated by cyclic voltammetry (CV) and galvanostatic charge-discharge cycling in 1 M NaPF_6_ in a mixed solvent of ethylene carbonate (EC) and diethyl carbonate (DEC). Figure 4a shows the CV curves of the TiO_2_@C electrode at the scan rate of 0.5 mV s^−1^. During the first cathodic scan, a large and broad reduction band appears at the potential region from 1.5 to 0 V, which considerably decreases its intensity during subsequent cycles, suggesting the formation of solid electrolyte interface (SEI) by decomposition of electrolyte. In the subsequent scans, a pair of redox peaks located at 0.7 and 0.85 V, referring the Na^+^ insertion/extraction reactions in the host lattice of anatase TiO_2_. It is noteworthy that the cathodic and anodic currents exhibit a gradual increase in the first ten cycles, possibly ascribed to an activation process of the TiO_2_@C electrode.

Figure 4b shows the typical charge/discharge profiles of the TiO_2_@C electrode at the current density of 0.05 A g^−1^. In accordance with the CV curves, the TiO_2_@C electrode demonstrates sloping charge/discharge profiles in the potential range of 0.3–1.3 V (vs Na/Na^+^). The initial charge and discharge capacities of TiO_2_@C are 649 and 317 mAh g ^−1^ (based on the weight of TiO_2_@C composite), corresponding to an initial columbic efficiency of 48.9%. The irreversible capacity during the first several cycles is due to the formation of SEI film by electrolyte decomposition and some form of irreversible trapping of Na^+^ in the TiO_2_ lattice. As shown in Fig. 4c, the reversible capacities of the TiO_2_@C remain stably at 298 mA h g^−1^ over 100 cycles, suggesting an outstanding cycling stability. For comparison, the TiO_2_ deliver a much lower reversible capacity of less than 100 mAh g^−1^ with an inferior initial columbic efficiency of 28% (Fig. S5). Considering the possible capacity contribution of the carbon additives, we also measured the Na-storage capacities of the acetylene black (Fig. S6). The capacity contribution of the carbon additive is calculated to be to be less than 10 mAh g^−1^, which is negligible. It is noteworthy that the reversible capacity of TiO_2_@C is possibly one of the highest capacities reported for the TiO_2_ anodes^23^,^26^,^28^.

In addition to the remarkable high capacity, the TiO_2_@C electrode also exhibits superior high rate capability and long-term cycling stability. Figure 4d compares the rate capability of the TiO_2_ and TiO_2_@C electrodes. The TiO_2_@C electrode delivers a reversible capacity of 311.5, 277.8, 257, 235, 214.6, 181.8, 125.5, 91.3 mAh g^−1^ at different current densities of 0.05, 0.1, 0.2, 0.4, 0.8, 1.6, 3.2 and 6.4 A g^−1^, respectively. More encouragingly, after cycled at different current densities for 90 cycles, the TiO_2_@C electrode recovers a reversible capacity of 289 mAh g^−1^ when the current density returns back to 0.05 A g^−1^, about 93.2% of its initial capacity. In contrast, the TiO_2_ electrode shows much poor rate performances and can only deliver a reversible capacity of less than 70 mAh g^−1^ at the current density of 1.6 A g^−1^, indicating a significant enhancement in rate capability of the TiO_2_ after carbon coating. In order to further evaluate the long-term cycling stability of the TiO_2_@C, cells are assembled and galvanostatically charged and discharged at 0.4 A g^−1^ for 500 cycles. As shown in Fig. 4e, a reversible capacity of 241 mAh g^−1^ is obtained after 500 cycles with a capacity retention of 85.2%. The coulombic efficiency rapidly rise up to 99.2% in the first few cycles, indicating stable reversibility.

The excellent electrochemical performance of TiO_2_@C can be ascribed to the synergistic effect of the well-dispersed TiO_2_ nanoparticles and the homogeneous carbon coating. The nanostructured TiO_2_ are beneficial for Na storage on account of the large surface areas, short diffusion length and fast kinetic properties. The surrounding carbon matrix, derived from the polyethyleneglycol, can not only provide abundant active sites for Na^+−^insertion/desertion, but also offers high electric conduction paths for fast electron transport, leading to a remarkable reversible capacity and strong rate capability. Moreover, the uniform carbon layer can stabilize the SEI formation by preventing the TiO_2_ nanoparticles from aggregating and attacking by electrolyte, thus resulting in high initial coulombic efficiency and long cycle life.

To further provide a better understanding of the improved electrochemical performance by carbon coating, electrochemical impedance spectra (EIS) of the TiO_2_@C and TiO_2_ electrodes are obtained in the frequency range from 100 KHz to 0.1 Hz. As shown in Fig. S7, the semicircles in the high-frequency region is attributed to the interface reaction of SEI film, while the medium-frequency semicircle is assigned to the real axis corresponding to the sodium-diffusion process in the bulk phase. The TiO_2_@C electrode exhibits much lower SEI film resistance (R_SEI_, 13.3 Ω) and charge transfer resistance (R_ct_, 305.4 Ω) than those of the TiO_2_ electrode (87.2 Ω and 403.9 Ω) based on the equivalent circuit simulation, respectively, indicating better electronic and ionic conduction in the TiO_2_@C composite.

## Conclusion

In summary, we present a convenient and green one-pot solvothermal method to fabricate carbon-coated TiO_2_ nanoparticles (TiO_2_@C). The ethylene glycol serve as both the reaction mediate and carbon source without adding any other carbon additives. Benefiting from the well-dispersed nanoparticles and homogeneous carbon coating, the as-prepared TiO_2_@C demonstrate a high reversible capacity of 317 mAh g^−1^, strong rate capability of 125 mAh g^−1^ at 3.2 A g^−1^ and superior cycling stability over 500 cycles, offering a low cost and high performance anode material for Na-storage. Particularly, the synthesis route described in this work is simple and intrinsically green, which provide new insights for the development of better host materials for practical SIBs.

## Methods

### Material Synthesis

The carbon-coated TiO_2_ nanoparticles were synthesized by solvothermal process as schematically illustrated in Fig. 1. Typically, 1 ml TiCl_4_ were added into 80 ml ethylene glycol dropwise with continuous stirring until the solution became clear. Then 2 ml ammonium hydroxide (25%) were added into the above solution and stirred for another 15 mins. The mixed solution was transferred into a 100 ml Teflon-lined stainless steel autoclave and treated at 180 °C for 24 h. After cooling down to room temperature, the products were collected by centrifugation and washed several times with ethanol and distilled water. Then the samples were dried at 80 °C under vacuum for 10 h to obtain the raw powders of TiO_2_ (denoted as TiO_2_-raw). This raw material was then calcined in Argon atmosphere at 700 °C for 2 h to obtain the carbon-coated TiO_2_ nanoparticles (denoted as TiO_2_@C). For comparison, TiO_2_ nanoparticles were also prepared in the same way as above except for annealing in air (denoted as TiO_2_).

### Material characterization

The crystalline structure of the as-prepared materials were recorded on powder X-ray diffraction (XRD, PANalytical B.V., Holland) using Cu-Kα radiation. Particle morphologies were characterized by scanning electron microscopy (SEM, SIRION200) and transmission electron microscopy (TEM, JEOL2100). Fourier transformed infrared (FTIR) spectra were recorded on a Bruker VERTEX 70 FTIR spectrometer. Raman spectroscopic analysises were performed with a Horiba Jobin-Yvon LabRAM HR800 Raman system using laser excitation at 532 nm from an Nd-YAG laser. The specific surface area were determined by Brunauer–Emmett–Teller (BET) nitrogen adsorption–desorption measurement on TriStar II 3020. X-ray photoelectron spectroscopic (XPS) measurement was performed on an AXIS-ULTRA DLD X-ray photoelectron spectrometer. Carbon content of the carbon-coated anatase-phase TiO_2_ was confirmed by TG-DSC (Netzsch STA 449 F5) in an air atmosphere with a heating rate of 10 °C/min from room temperature to 800 °C.

### Electrochemical measurements

The working electrodes were prepared by mixing the TiO_2_@C (TiO_2_), acetylene black and polyvinylidene difluoride (PVDF) in N-methyl-2-pyrrolidine (NMP) in a mass ratio of 80: 10: 10. The slurry was coated uniformly (doctor-blade) on Cu foil and vacuum-dried at 110 °C for more than 12 h. Electrochemical tests were carried out using CR2016 coin cells, which were assembled in glove box filled with highly pure argon gas (O_2_ and H_2_O levels <0.1 ppm). Sodium metal acted as the counter and reference electrode, Celgard 2400 membrane as the separator. The electrolyte was 1 M NaPF_6_ salt in a mixture of ethylene carbonate (EC) and dimethyl carbonate (DEC) solution (EC: DEC, 1:1 in volume) with the addition of 10 wt% fluoroethylene carbonate (FEC). Cyclic voltammetry (CV) was measured on an electrochemistry workstation (CHI 660E) using a scan rate of 0.5 mV s^−1^. Galvanostatic discharge/charge cycling was made on a LANHE battery test system (Wuhan, China) in the voltage range of 0 ~3 V (vs. Na/Na^+^). Electrochemical impedance spectroscopy (EIS) analysis was conducted using an electrochemical workstation (Autolab, PGSTAT302N) with the frequency range of 100 kHz to 0.1 Hz after operating the electrodes for 100 cycles.

## Additional Information

**How to cite this article**: Tao, H. *et al*. Glycol Derived Carbon- TiO_2_ as Low Cost and High Performance Anode Material for Sodium-Ion Batteries. *Sci. Rep.* **7**, 43895; doi: 10.1038/srep43895 (2017).

**Publisher's note:** Springer Nature remains neutral with regard to jurisdictional claims in published maps and institutional affiliations.

## Supplementary Material

Supplementary Information
