# Supplementary material for: Glycol Derived Carbon- TiO2 as Low Cost and High Performance Anode Material for Sodium-Ion Batteries
Source: Sci Rep. 2017 Mar 3;7:43895. doi: 10.1038/srep43895 (PMC5335553; doi:10.1038/srep43895)
Supplement: Supplementary Information [file srep43895-s1.pdf]

Supporting Information

Title: Glycol Derived Carbon- TiO_2 as Low Cost and High Performance Anode Material for Sodium-Ion Batteries

Hongwei Tao†, Min Zhou†, Kangli Wang*, Shijie Cheng, Kai Jiang*

† These authors contributed equally to this work

1. FTIR-Spectra of TiO_2-raw, TiO_2@C and TiO_2

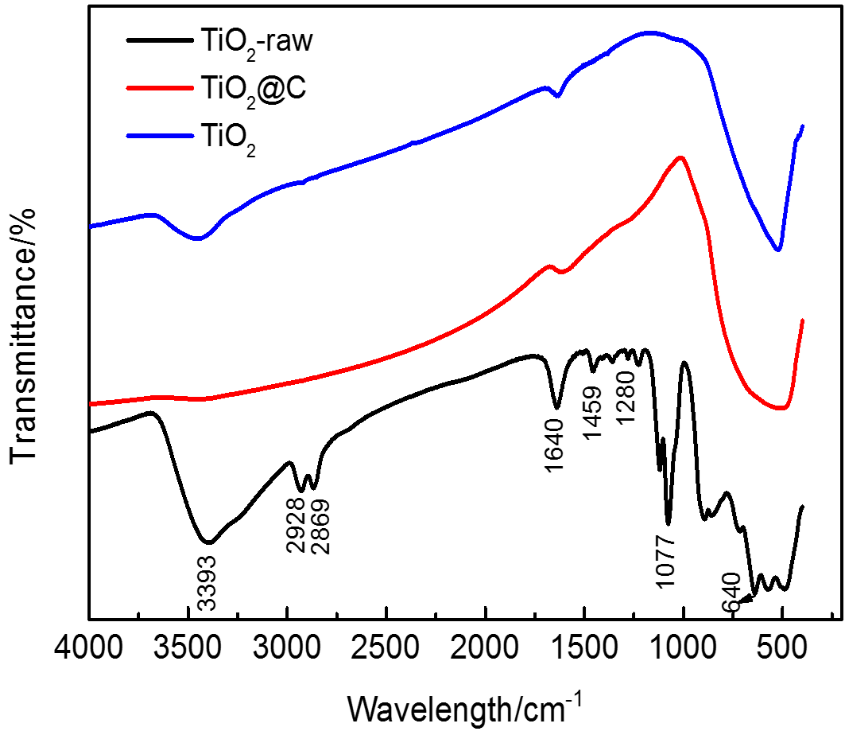

Fig. S1 FTIR-Spectra of TiO_2-raw, TiO_2@C and TiO_2

The FT-IR spectrum of the TiO_2-raw material reflects all the characteristic absorptions of the typical precursor $Ti[(OCH_2CH_2)_nO]_2$. The peaks of 3393 cm^{-1} and 1640 cm^{-1} are assigned to the stretching vibrations of O-H, corresponding to the adsorbed water.[1,2] The bands located at 2928 cm^{-1} and 2869 cm^{-1} belong to the stretching vibrations of C-H.[3-6] The peak at 1459 cm^{-1} is assigned to O-CH_2 bending vibration.[3-5] Three peaks at 1359 cm^{-1}, 1280 cm^{-1} and 1226 cm^{-1} can be attributed to C-H wagging and twisting vibration, respectively. [3-5] The peak at around 1119 cm^{-1} and 1077 cm^{-1} is assigned to the C-O and C-O-C stretching vibration, indicating the formation of polyethyleneglycol.[3-5] The bands at 640 cm^{-1} and 575 cm^{-1} can be assigned to Ti-O-C, while the band at 490 cm^{-1} is the characteristic of the Ti-O-Ti stretching and bending vibration, indicating the coordination of the polymer with Ti^{4+}.[6] All the adsorption bands stated above suggest the formation of the polymeric ligand, which pyrolize and convert to carbon coated TiO_2 particles in the subsequent annealing process.

2. X-ray photoelectron spectroscopic (XPS) spectra of TiO_2@C.

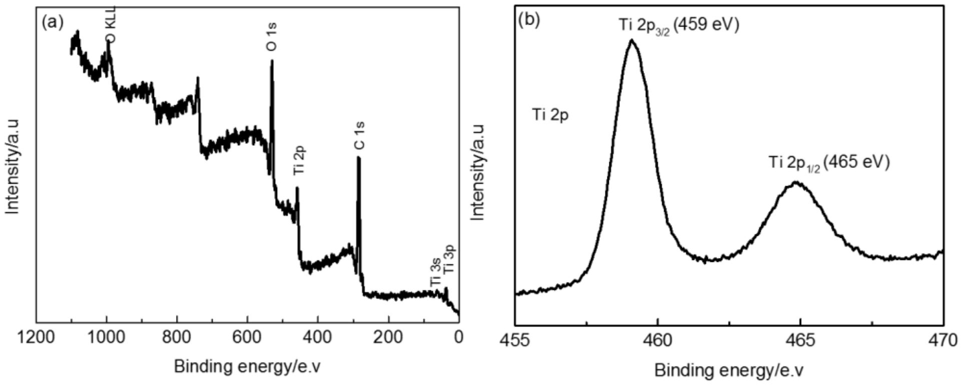

Fig. S2 Typical XPS spectra of the TiO_2@C: (a) survey spectra, (b) Ti 2p region XPS spectrum.

3. The photo images of the TiO_2@C and TiO_2.

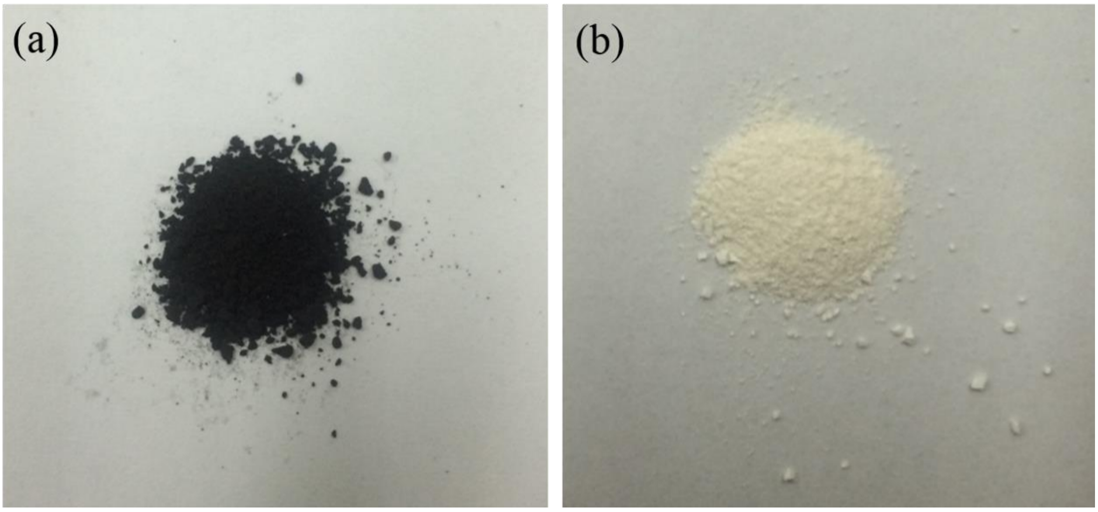

Fig. S3 The photo images of the (a)TiO_2@C and (b)TiO_2.

4. **TEM images of TiO_2.**

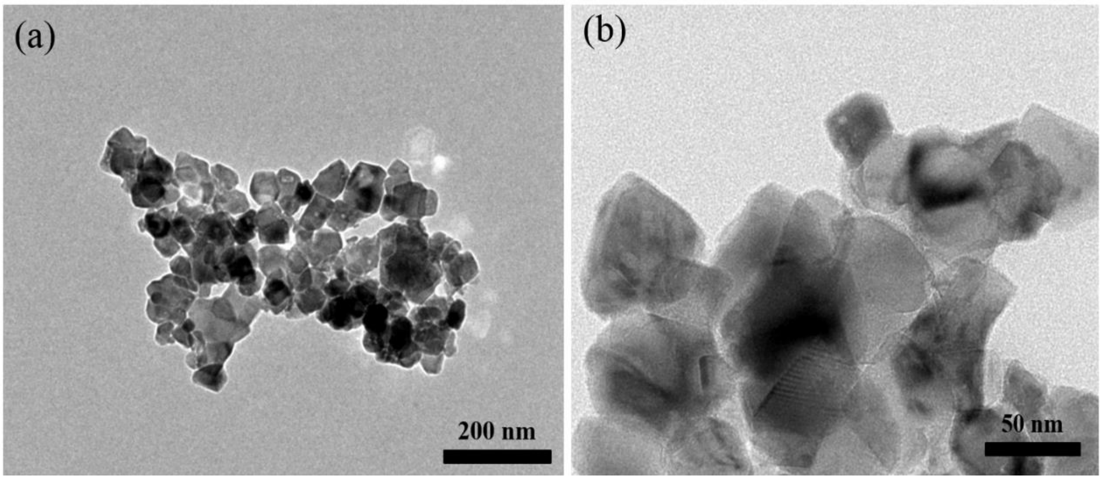

Fig.S4 TEM images of TiO_2: (a) Low and (b) high magnification.

5. **The electrochemical performance of TiO_2 electrode**

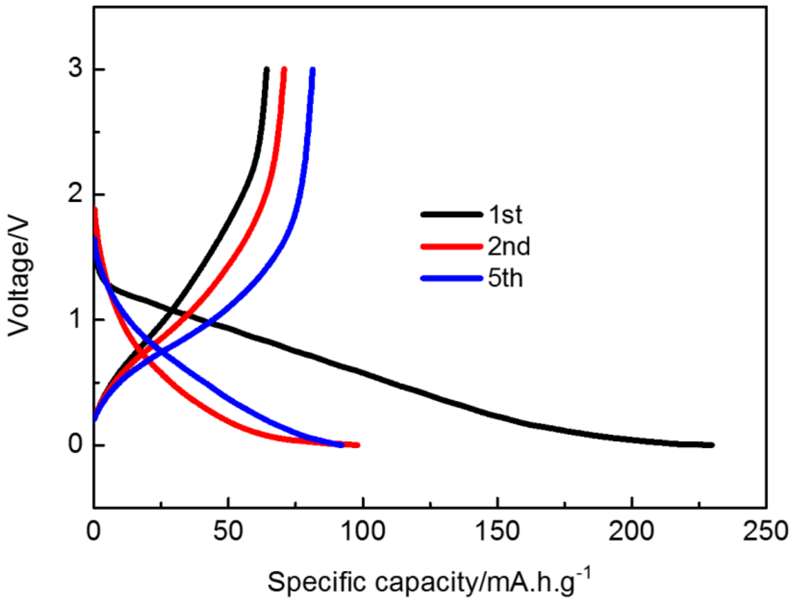

Fig. S5. The charge-discharge profiles of TiO_2 electrode in 1.0 mol L^{-1} $NaPF_6$ +EC-DEC (v/v=1/1) electrolyte at the current density of 0.05 A g^{-1} in a voltage range of 0 to 3 V.

6. **The electrochemical performance of carbon additives (acetylene black)**

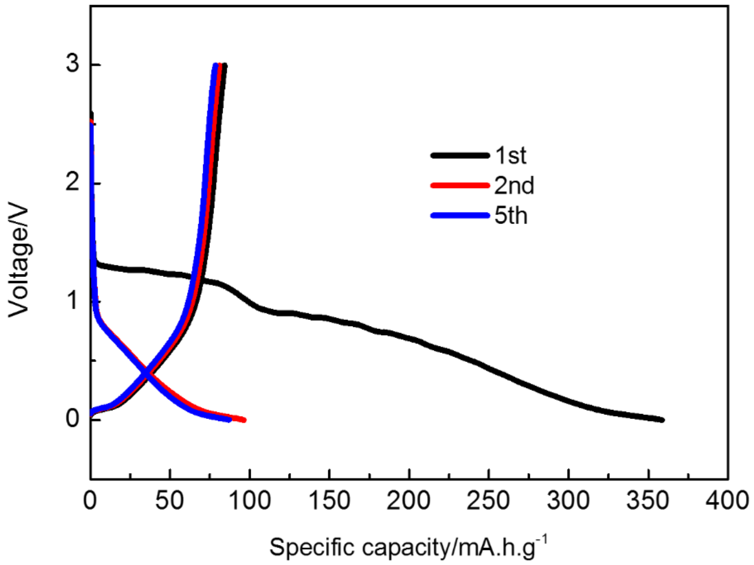

Fig. S6.The charge-discharge profiles of acetylene black (AB) in 1.0 mol L^{-1} $NaPF_6$ +EC-DEC (v/v=1/1) electrolyte at the current density of 0.05 A g^{-1} in a voltage range of 0 to 3 V.

As shown in Fig. S3, the carbon addictive acetylene black (AB) can deliver a reversible capacity of 76.8 mAh g^{-1} at the current densities of 0. 05 A g^{-1}. As the weight ratio of TiO_2/AB=8:1, the capacity contribution of the carbon addictive is calculated to be 9.6 mAh g^{-1}.

7. Electrochemical impedance spectra (EIS) of the TiO_2@C and TiO_2 at fully charge state after 100 cycles.

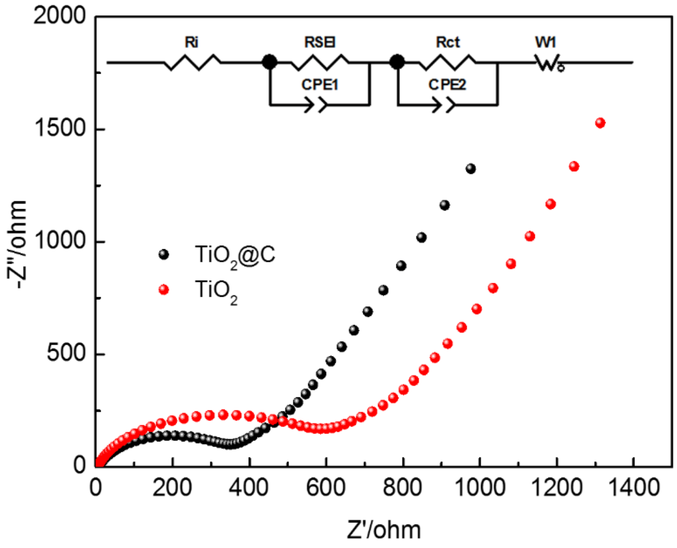

Fig. S7. Electrochemical impedance spectra (EIS) of the (a) TiO_2@C and (b) TiO_2 at fully charge state after 100 cycles.

Table S1 Fitting results of the Nyquist plots using the equivalent circuit.

samples	R_i (Ω)	R_{SEI} (Ω)	CPE_1(F)	R_{ct}(Ω)	CPE_2(F)	Chi-Squared

TiO_2@C	3.2	13.3	1.4×10^{-5}	305.4	8.0×10^{-5}	1.0×10^{-3}
TiO_2	3.3	87.2	1.5×10^{-3}	403.9	1.5×10^{-5}	1.0×10^{-3}

Reference

1 Mansur, H. S., Oréfice, R. L. & Mansur, A. A. Characterization of poly (vinyl alcohol)/poly (ethylene glycol) hydrogels and PVA-derived hybrids by small-angle X-ray scattering and FTIR spectroscopy. *Polymer* **45**, 7193-7202 (2004).

2 Zhang, Y., Kohler, N. & Zhang, M. Surface modification of superparamagnetic magnetite nanoparticles and their intracellular uptake. *Biomaterials* **23**, 1553-1561 (2002).

3 Brubach, J. *et al.* Structural and thermal characterization of mono-and diacyl polyoxyethylene glycol by infrared spectroscopy and X-ray diffraction coupled to differential calorimetry. *The Journal of Physical Chemistry B* **108**, 17721-17729 (2004).

4 Evans, C. C., Bates, F. S. & Ward, M. D. Control of hierarchical order in crystalline composites of diblock copolymers and a molecular chromophore. *Chemistry of materials* **12**, 236-249 (2000).

5 Jeevanandam, P. & Vasudevan, S. Intercalation of Alkali Metal-Polyethylene Oxide Polymer Electrolytes in Layered CdPS3. *Chemistry of materials* **10**, 1276-1285 (1998).

6 Wang, D., Yu, R., Kumada, N. & Kinomura, N. Hydrothermal synthesis and characterization of a novel one-dimensional titanium glycolate complex single crystal: Ti $(OCH_2CH_2O)_2$. *Chemistry of materials* **11**, 2008-2012 (1999).
